# Humoral response dynamics following inactivated SARS-CoV-2 vaccination and their association with subsequent infection and symptoms in individuals with and without prior SARS-CoV-2 infection: evidence from Sichuan Province, China

**DOI:** 10.1128/spectrum.02191-24

**Published:** 2025-08-11

**Authors:** Yunhan Yu, Mengmeng Jia, Jingchuan Zhong, Luzhao Feng, Xiaoman Jiang, Yanan Li, Qiangru Huang, Yuezhu Chen, Xinming Wang, Yong Yue, Li Guo, Xian Liang, Lili Ren, Weizhong Yang

**Affiliations:** 1 Chengdu Workstation for Emerging Infectious Disease Control and Prevention, Chinese Academy of Medical Science, Chengdu, China; 2 Chengdu Center for Disease Control and Prevention 605337, Chengdu, China; 3 Vanke School of Public Health, Tsinghua University 12442, Beijing, China; 4 National Institute of Pathogen Biology, Chinese Academy of Medical Sciences & Peking Union Medical College, Beijing, China; 5 School of Population Medicine and Public Health, Chinese Academy of Medical Sciences & Peking Union Medical College, Beijing, China; 6 Key Laboratory of Pathogen Infection Prevention and Control (Ministry of Education), State Key Laboratory of Respiratory Health and Multimorbidity, National Institute of Pathogen Biology, Chinese Academy of Medical Sciences & Peking Union Medical College, Beijing, China; 7 NHC Key Laboratory of Systems Biology of Pathogens and Christophe Mérieux Laboratory, National Institute of Pathogen Biology, Chinese Academy of Medical Sciences & Peking Union Medical College, Beijing, China; 8 Chengdu High-Tech Zone Center for Disease Control and Prevention, Chengdu, China; 9 Key Laboratory of Respiratory Disease Pathogenomics, Chinese Academy of Medical Sciences & Peking Union Medical College, Beijing, China; National Taiwan University, Taipei, Taiwan

**Keywords:** humoral immunity, inactivated vaccination, reinfection, trajectory analysis, dynamic immune patterns

## Abstract

This study integrated multisource longitudinal data with trajectory modeling to delineate the heterogeneity of humoral immune dynamics induced by inactivated SARS-CoV-2 vaccinations and their clinical implications for subsequent SARS-CoV-2 infection outcomes in 205 individuals from Sichuan Province, China. We found that preexisting infection status served as the dominant stratifier of antibody trajectory divergence across the cohort. Additionally, among individuals without prior infection before the vaccine cohort, we identified five distinct immune response patterns with clinical implications. An age-associated “minimal response” subtype was associated with an increased risk of prolonged recovery time, sore throat, and limb pain in subsequent infections. In contrast, a subtype characterized by a marked increase in S-Igs titers after the booster dose exhibited fewer symptoms, with a lower likelihood of experiencing fever and fatigue. What is more, for those with prior infection, clinical data—including viral shedding duration and total antibody levels 15 days after discharge from the initial infection—could provide valuable insights into the subsequent risk of reinfection.

**IMPORTANCE:**

To better identify vulnerable populations in epidemic surveillance and predict their clinical manifestations post-infection for accurate diagnosis and effective management, it is vital to understand the intrinsic dynamic immune patterns among individuals and how these trajectory patterns relate to future infections and associated symptoms. In the post-COVID-19 era, conducting nuanced analyses remains of great significance, especially as longer-term observational data become available. This is the first finding from China that illustrates the dynamic characteristics of the immune response following inactivated COVID-19 vaccination over an extended observation period, including information on following infection and symptoms. These data are particularly valuable as no participants experienced COVID-19 infection during the vaccine cohort follow-ups, meaning their antibody levels solely reflect the intrinsic dynamic immune patterns triggered by the inactivated COVID-19 vaccines.

## INTRODUCTION

As of January 14, 2024, more than 774 million confirmed cases and over 6 million associated deaths have been attributed to the significant threat posed by the severe acute respiratory syndrome coronavirus 2 (SARS-CoV-2) to global public health (1). Despite its containment no longer being a priority for the current global public health agenda, SARS-CoV-2, as the most influential global infectious disease of modern times, has generated invaluable data that will be highly significant for the future surveillance, management, and diagnosis of infectious diseases.

Understanding infection-induced and vaccine-induced SARS-CoV-2 humoral immunity through serological assays provides critical insights into immune persistence mechanisms and evidence-based vaccination policies (2). While prior studies have mapped antibody evolution in convalescent patients across symptom strata (3–6), research on vaccine-induced responses remains predominantly focused on efficacy comparisons with natural infection (7–11), leaving population-level heterogeneity in dynamic antibody trajectories insufficiently characterized (12, 13). Although trajectory modeling offers a methodological framework for capturing humoral response dynamics (5, 6), the unknown history of natural infection in the general population has introduced significant confounding effects on trajectory classification in previous studies of vaccine-induced immunity (14, 15). Filling this research gap is essential, as investigating vaccine-induced antibody dynamics, particularly in individuals without prior infection, can help identify distinct humoral response trajectory subtypes influenced by factors such as age, sex, comorbidities, and other biological traits (such as genetic factors) (12, 13, 16–22), ultimately guiding the development of personalized vaccination strategies.

Moreover, while previous studies have demonstrated the significant impact of both the dynamics of extrinsic factors, such as age and comorbidities, and intrinsic factors, such as immune response, on clinical outcomes during SARS-CoV-2 infection and reinfection with new variants (23), the existing evidence primarily focuses on infection-induced rather than vaccine-induced immune responses (19, 24). However, it is also of great significance to examine whether the dynamics of vaccine-induced humoral responses can predict subsequent infection status and associated symptoms, as this insight could facilitate the identification of at-risk subgroups and enhance preparedness for future medical resource allocation during pandemics. Therefore, bridging this gap requires longitudinal monitoring that encompasses both the early phases of vaccine-induced immune responses and the later stages of durability assessment.

To address existing evidence gaps, we conducted an extensive multisource analysis in Sichuan Province, China. This analysis was based on a longitudinally validated vaccine cohort data set (April 2021–July 2022) and supplemented with a telephone follow-up in July 2023 to assess subsequent infections and associated symptoms—primarily occurring during the Omicron-dominant waves—following the initial cohort period. Additionally, we incorporated clinical data, including follow-up information obtained 15 days post-discharge, from individuals with prior infections during their initial infection.

For the vaccine cohort, enrollment was conducted between April 2021 and July 2021. Despite the absence of local transmission in Chengdu during this period, genomic surveillance data from imported cases and neighboring provinces confirmed the dominance of the Delta variant (B.1.617.2) in China (Chinese CDC Weekly, 2021). All participants in the cohort received inactivated vaccines based on the ancestral strain (CoronaVac/Sinopharm) and participated in vaccine follow-ups in the subsequent year. These vaccines demonstrated 65.9% (95% CI: 65.2–66.6) effectiveness against Delta variant symptomatic infection and 87.5% (86.7–88.2) protection against hospitalization in contemporaneous trials (25). Notably, there were zero breakthrough infections during the vaccine cohort, avoiding the trajectory data being confounded by natural infection during the vaccine cohort. This makes the data set a unique resource for isolating vaccine-induced humoral dynamics from the confounding effects of unknown natural infection status.

The key objectives of our investigation are to identify distinct time-dependent immune subtypes using trajectory modeling, to understand whether they are influenced by demographic factors and health behaviors, and to determine whether these extrinsic and intrinsic factors can predict subsequent infection outcomes during the Omicron-dominant waves.

## RESULTS

A total of 205 participants from Sichuan Province, consisting of 39 individuals with prior SARS-COV-2 infection and 166 individuals without prior SARS-CoV-2 infection, were enrolled in this vaccination cohort study in 2021. Among the 205 individuals, the median age was 46, with almost half being female (50.7%). There were no significant differences in age, sex proportion, drinking, and smoking status between the two groups. However, differences were observed in education level and the presence of noncommunicable diseases (NCD) (Table S1). Throughout the one-year follow-up period, none of the respondents was infected with SARS-CoV-2. Those lost to follow‐up were mainly due to interregional population mobility.

Of the 205 respondents in the study, 177 participated in the telephone follow-up conducted in July 2023, comprising 145 previously uninfected and 32 previously infected individuals. Statistical analyses revealed no significant demographic differences between those who completed the follow-up and the overall study cohort (Table S21).

Among these 177 participants, 149 were infected during the subsequent year after the vaccine cohort. Of these, 118 individuals were infected in December 2022 (79.2%), and 140 (94.0%) were infected between November 2022 and January 2023. The close proximity in time and location of infections ensured comparability across different respondents. Self-reported infection status and the associated symptoms were collected. Results revealed that 127 people (87.6%) who were not previously infected contracted SARS-CoV-2, while 17 people (53.1%) with a history of infection also tested positive. The close proximity in time and location of infections ensured comparability across different respondents. No significant correlations were found between the associated symptoms in later infection, as all Phi coefficients had absolute values less than 0.20 (Table S20).

### Antibody titers of individuals with and without natural infection at each follow-up

**Fig 1:**
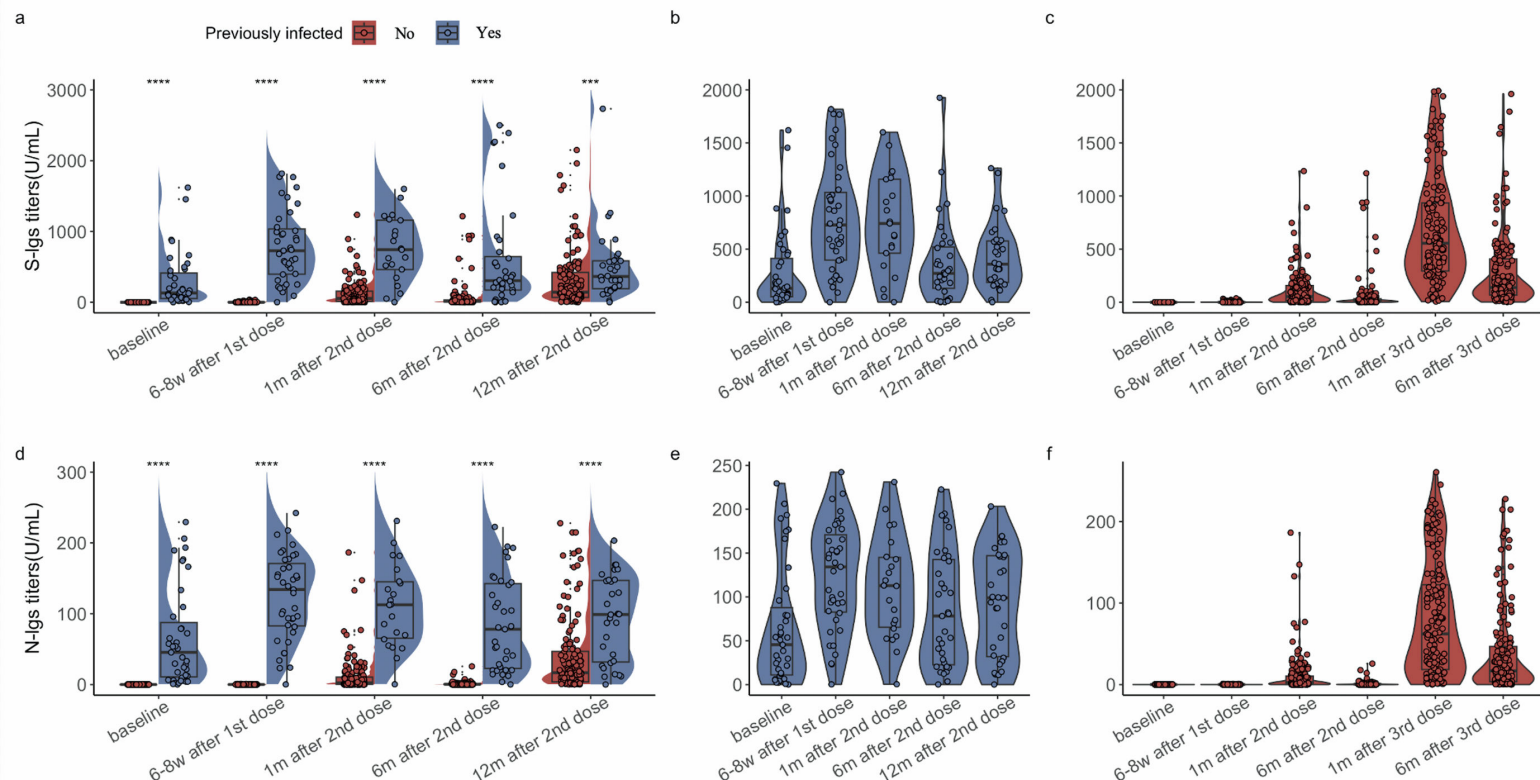
Titers of S-Igs and N-Igs during all follow-up visits (*N* = 205). (a) and (d) are box plots and violin plots of S-Igs and N-Igs, respectively, among individuals with and without prior infection at enrollment in the vaccine follow-ups. The * above the plots indicate the significance levels of the *t*-test comparing the average antibody levels detected during each follow-up between the two groups. *** *P*-value < 0.001 and **** *P*-value < 0.0001. (b) and (e) are box plots and violin plots of S-Igs and N-Igs, respectively, among individuals with prior infection at enrollment for each vaccine follow-up they participated in. (c) and (f) are box plots and violin plots of S-Igs and N-Igs, respectively, among individuals without prior infection at enrollment for each vaccine follow-up they participated in.

**Fig 2:**
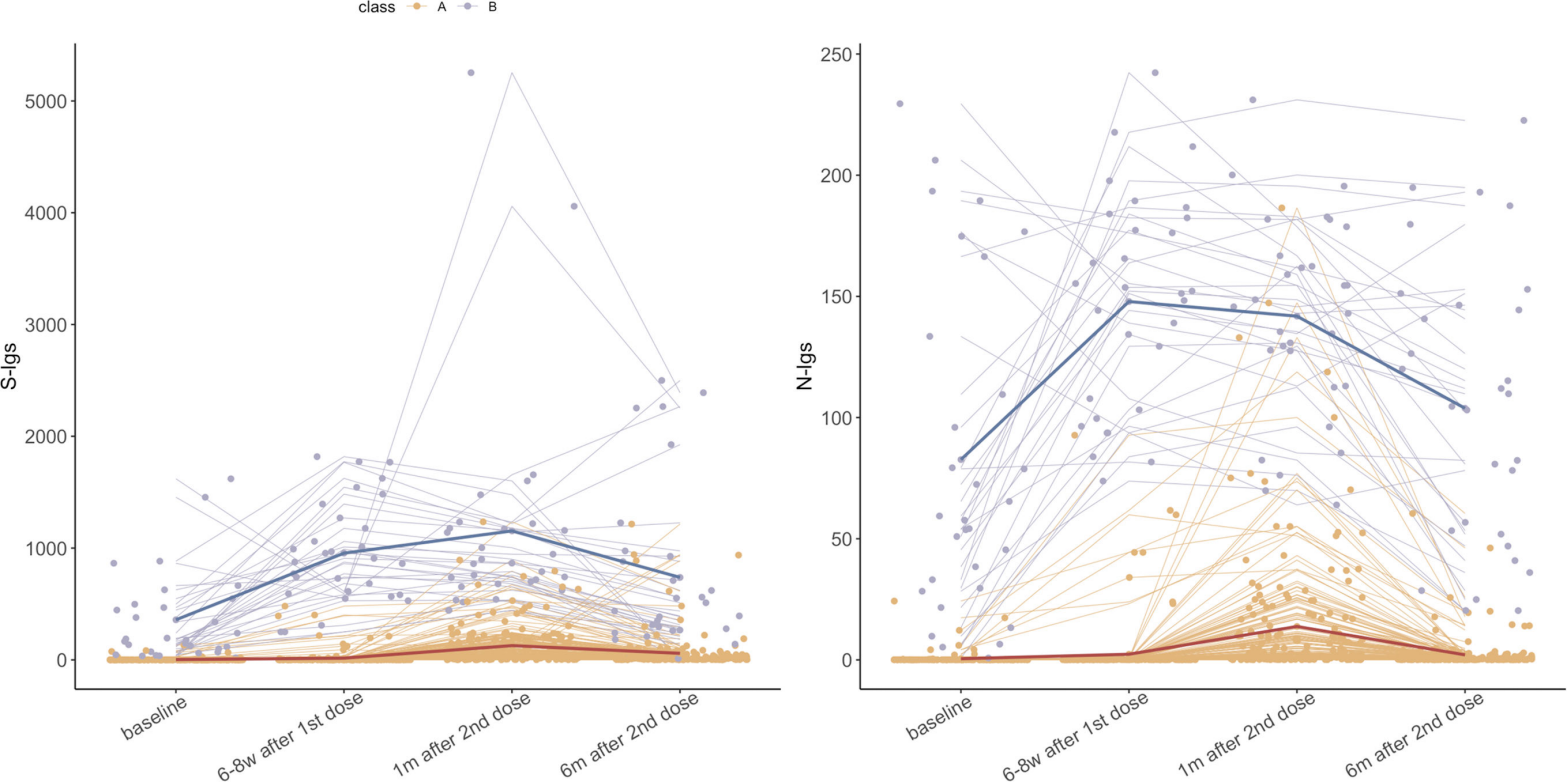
Two trajectory classes were identified from all individuals using a nonparametric method (*N* = 205). The bold line represents the average trajectories of the two classes. Other lines depict individual trajectories, with colors indicating the class to which they are assigned.

Among the SARS-CoV-2 naïve individuals, the baseline levels of S‐Igs or N‐Igs were undetectable. Increased levels of both S‐Igs and N‐Igs were observed six weeks after their first dose, with a more pronounced increase observed one month after the second dose. In contrast, individuals with natural infection displayed discernible titers, and a significant elevation was noted six weeks after their first dose. This heightened level was sustained, maintaining a similar magnitude until one month after the second dose. The titers of S-Igs and N-Igs in both groups declined over the subsequent five months. However, individuals with natural infection managed to achieve an increase compared to baseline (*P* value < 0.001): from a median of 131.90 (IQR: 53.05–412.45) to a median of 305.40 (IQR: 172.00–643.10) for S-Igs and from a median of 45.43 (IQR: 10.99–87.63) to a median of 78.18 (IQR: 22.64–142.55) for N-Igs (Fig. 1). In the initial four follow-up visits, the average levels of S‐Igs or N‐Igs are significantly higher in individuals with natural infection compared to naïve individuals (Fig. 2). A few naïve individuals showed comparable antibody titers as individuals with natural infection one month after the second dose.

Naïve individuals underwent an additional follow-up after receiving a booster vaccination six months after the second dose, which occurred after the fourth follow-up. A stronger humoral immune response was observed one month after booster vaccination, and the antibody titers remained at a relatively high level six months after the booster dose compared to one month after the second dose (from median: 53.53 [IQR: 14.59–156.45] to median: 144.70 [IQR: 68.48–432.05] for S-Igs; from median: 2.38 [IQR: 0.34–10.37] to median: 16.78 [IQR: 3.62–46.82] for N-Igs) (Fig. 2).

### Antibody trajectories pattern and effects of demographic variables among all participants

**Fig 3:**
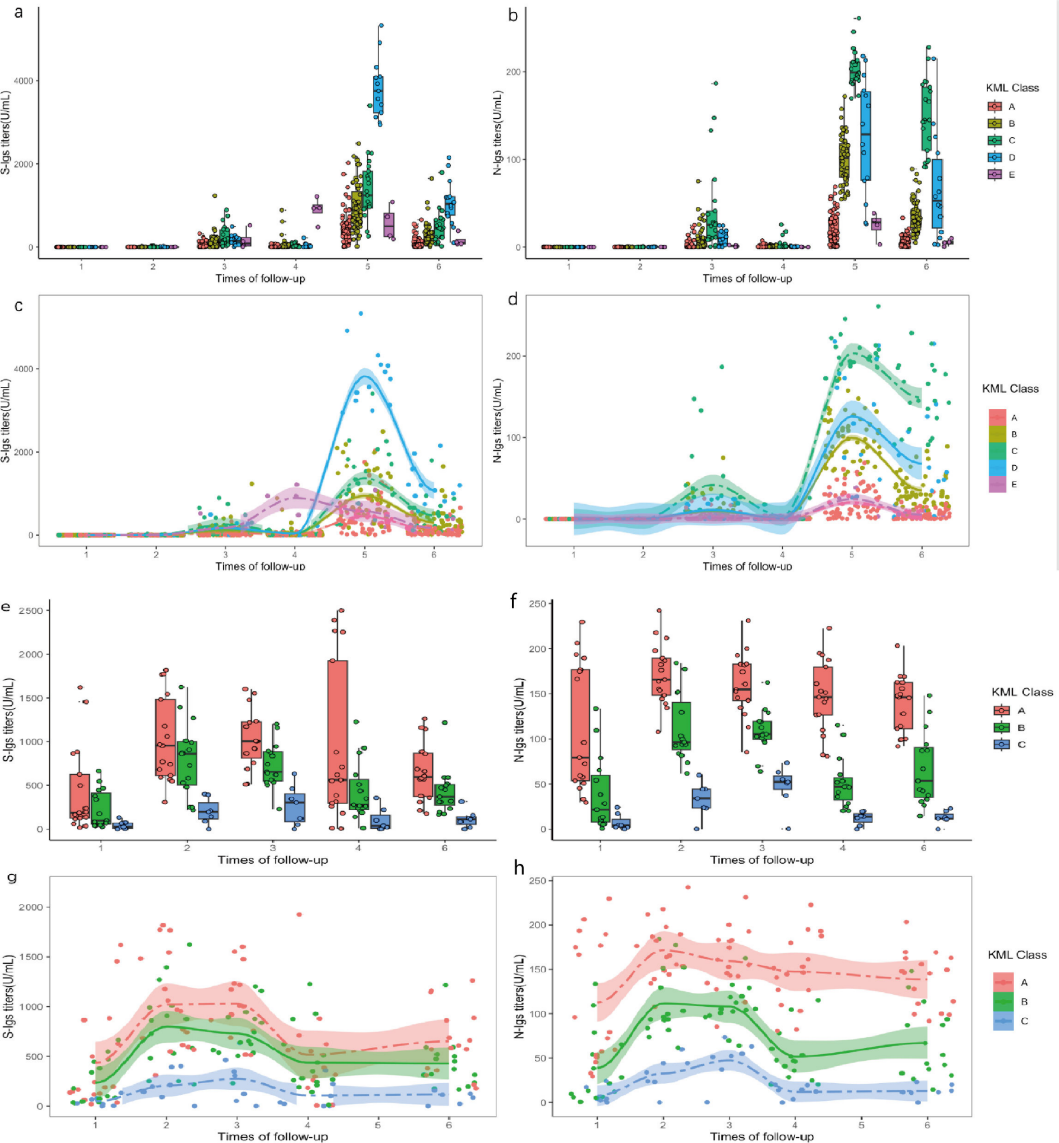
Box plot and mean trajectory of each class for individuals with and without prior infection. (a) and (b) were box plots of S-Igs and N-Igs for five classes identified among individuals without prior infection, and (c) and (d) were the mean trajectories corresponding to (a) and (b). (e) and (f) were box plots of S-Igs and N-Igs for three classes identified among individuals with prior infection, and (g) and (h) were the mean trajectories corresponding to (e) and (f); for (a)–(d): *N* = 166, and for (e)–(h): *N* = 39.

Two K-means Longitudinal (KML) classes were identified using data from the initial four follow-ups of all participants according to the distance of trajectories without adjusting other variables (Fig. 3; Fig. S2). Trajectories in class A exhibited low antibody titers, with a peak occurring one month after the second dose, while trajectories in class B began at a certain baseline level with more noticeable growth. Previous infection was identified as a key indicator for class membership (Table S2). The two distinct patterns identified through clustering closely align with the trajectories of the two groups of individuals stratified by prior infection (Fig. 2; Fig. S3). A χ test revealed a significant association between KML class membership and prior infection status (*P* < 0.001) ( Table S2). And all the naïve individuals and nine individuals with prior infection were assigned to class A, while 30 individuals with prior infection were assigned to class B.

Growth mixture model obtained a similar classification, assigning infection status as the predictor of class membership, assigning two classes, and adjusting for extrinsic characteristics (demographic and health-related factors). The fixed effects in the longitudinal model suggested that individuals with diabetes and females tend to have a higher level of S-Igs titers, and individuals with older age tend to have a lower level of N-Igs titers (Table 1).

**TABLE 1:** Fixed effects of individuals‘ extrinsic characteristics on the two humoral response trajectories identified among all individuals^*a*^

		S-lgs			N-lgs		
Variable	Category	Estimate	95% CI	*P*-value	Estimate	95% CI	*P*-value
Age	/	−0.003	(−0.01, 0.002)	0.27	−0.006	(−0.01, −0.001)	0.02
Gender	Male	Ref			Ref		
	Female	0.22	(0.03, 0.40)	0.02	0.14	(−0.03, 0.31)	0.11
Diabetes	No	Ref			Ref		
	Yes	0.57	(0.24, 0.91)	<0.001	0.22	(−0.11, 0.56)	0.19
Recently smoke	No	Ref			Ref		
	Yes	0.03	(−0.20, 0.27)	0.78	0.12	(−0.12, 0.35)	0.32
Hypertension	No	Ref			Ref		
	Yes	0.08	(−0.20, 0.36)	0.58	0.12	(−0.16, 0.40)	0.4
Exposure to SHS	No	Ref			Ref		
	Yes	0.02	(−0.14, 0.17)	0.82	0.04	(−0.12, 0.20)	0.61
Recently drink	No	Ref			Ref		
	Yes	0.07	(−0.15, 0.29)	0.53	−0.01	(−0.23, 0.20)	0.91

^*a*^ SHS, secondhand smoke; Ref, reference group; /, Not applicable.

### Antibody trajectory patterns in individuals without prior infection at enrollment and their relationship to associated symptoms during initial infection

Among 166 individuals without prior SARS-CoV-2 infection at enrollment, the trajectory modeling techniques identified five distinct KML classes (Fig. 3a through d; Fig. S4), and the participants appeared to be reliably assigned to one of these classes, given that most of their class membership probabilities were high (Table S3). The results of the permutational multivariate analysis of variance (PERMANOVA) validated the significance of the differences in trajectories across the five identified KML classes (*P*-value: 0.001). Additionally, the KML classes accounted for a substantial proportion of the variation in composition (62.7%) (Table S18). Demographic factors, except age, show no difference in different KML classes (Table S4). After adjusting for age, no significant effects of vaccine type on trajectory clusters were observed at the 0.05 significance level (Table S17).

According to relevant records, none of the individuals without a prior infection at enrollment contracted the virus during the one-year follow-up period. However, the majority of those who responded to the additional telephone follow-up conducted one year after the vaccine follow-ups (132 out of 145) reported experiencing their initial SARS-CoV-2 infection. Since individuals who reported no infection until the telephone follow-up were distributed across different classes (Table S6), we focused on the correlation between the KML class of those who reported an initial infection during the telephone follow-up and their associated symptoms.

Class A (*N* = 53, 32%) comprises individuals for whom the vaccines elicited minimal increases in the levels of S-Igs and N-Igs titers, and who tend to demonstrate an older age profile compared to individuals in other classes (Table S5). After controlling for demographic factors and antibody titer test results in each follow-up using variable selection techniques, individuals in class A who went through SARS-CoV-2 infection were more likely to have a long recovery time (more than 15 days) (odds ratio: 14.50 [3.57, 58.99]), a sore throat (odds ratio: 14.50 [3.57, 58.99]), and pain in the limbs (odds ratio: 6.61 [1.13, 38.83]) (Table 2).

**TABLE 2:** Effects of individuals with no prior infection being in a particular trajectory class on symptoms in future initial infections (*N* = 145)^*a*^

Outcome	Metric	Class A	Class B	Class C	Class D	Class E
More symptoms	Coef	–^*b*^	–	−0.29 (−0.54, −0.04)	−0.44 (−0.79, −0.08)	–
Long recovery time	OR	14.50 (3.57, 58.99)	–	–	–	0.04 (0, 0.62)
Have fever	OR	–	–	–	0.01 (0, 0.39)	0.13 (0.01, 1.15)
Sore throat	OR	7.53 (1.75, 32.37)	–	–	–	–
Fatigue	OR	–	–	–	0.17 (0.04, 0.77)	–
Pain in the limbs	OR	6.61 (1.13, 38.83)	–	–	–	–

^*a*^ OR stands for odds ratio, and the corresponding model is logistic regression. Coef stands for coefficient, and the corresponding model is Poisson regression. Both the estimate and the 95% CI are shown in the table. Demographic and antibody titer variables adjusted for each model in Table 2 are shown in Tables S7 to 14.

^*b*^ "–” indicates that the class was not included in the model as a covariate and instead served as part of the reference group.

Class B (*N* = 39, 23%) exhibited a moderate elevation in S-Igs and N-Igs titers following booster dose (Fig. 3a through d). Consequently, this class was selected as the reference group in the analysis of the association between KML class and subsequent infection-related symptoms.

Meanwhile, class C (*N* = 38, 23%) demonstrated a significantly more pronounced increase in N-Igs titers following the booster dose compared to the other groups (Fig. 3a through d). Individuals in class C exhibited a lower incidence of symptoms upon infection (−0.29 [−0.54, −0.04]).

And as for class D (*N* = 21, 13%), it is notably characterized by a more substantial climb in S-Igs titers one month after the second dose of vaccination compared to other groups. They tended to have the least number of symptoms (−0.44 [−0.79, −0.08]) during the infection and were less likely to have fever (odds ratio: 0.01 [0, 0.39]) and fatigue (odds ratio: 0.17 [0.04, 0.77]).

Lastly, 15 participants (9%) were assigned to class E. Their S-Igs titers attained an exceptionally elevated level after the booster vaccination compared to individuals in other groups. However, N-Igs titers vary across individuals in class E (Fig. 3a through d). Individuals in class E had a lower risk of having a long recovery time (odds ratio: 0.04 [0.00, 0.62]).

### Antibody trajectories pattern among individuals with prior infection and predictors of their reinfection

Three KML classes with a similar trend at various levels were identified among individuals with prior infection (Fig. 3e thorugh h). But the results of the PERMANOVA suggested that the differences in trajectories across these three identified KML classes are not significant (Table S19). There were no significant differences among these three groups regarding demographic characteristics, clinical testing results during the initial infection, reinfection status, and occurrence of associated symptoms (Table S15.1 and S16).

As identifying individuals with an elevated risk of reinfection post-SARS-CoV-2 is crucial for formulating effective vaccination strategies, four predictors were selected (Table 3). It was observed that individuals with a longer duration of hospitalization or viral shedding had lower odds of reinfection (odds ratio: 0.627 [0.402, 0.979]). Conversely, the odds were higher for those exhibiting elevated total antibody levels 15 days after discharge. The correlation matrix for all included clinical variables (Fig. S1) showed a significant negative correlation between admission CD19+ count and total antibody levels 15 days after discharge. Thus, for those with lower admission CD19+ count, their risks of reinfection may be higher.

**TABLE 3:** Risk factors for SARS-COV-2 reinfection based on clinical data from the initial infection in previously infected individuals

Variable	OR	95% CI	*P*-value
Duration of hospitalization/viral shedding duration	0.627	(0.402, 0.979)	0.05
NK cell count 15 days after discharge	1.036	(0.997, 1.075)	0.08
Admission total antibody level	0.948	(0.888, 1.013)	0.13
Total antibody level 15 days after discharge	1.009	(1.001, 1.018)	0.04

Results obtained from the Fisher’s exact test (Table S15.2) indicate that there is no statistically significant difference in the likelihood of reinfection among individuals based on various factors, including KML class and other demographic variables.

The results may be attributed to the limited sample size resulting from the small number of individuals in Chengdu with a history of SARS-CoV-2 infection prior to the initiation of the cohort.

## DISCUSSION

This study integrated multisource longitudinal data with trajectory modeling to delineate the heterogeneity of vaccine-induced humoral immune dynamics and their clinical implications in SARS-CoV-2 infection outcomes. Collectively, these findings would better assist in identifying vulnerable populations during epidemic surveillance and managing epidemics more effectively.

The study by Wei et al. (14) employed latent class mixed models to analyze the dynamics of anti-spike antibody responses to natural SARS-CoV-2 infection at the population level. They found that uncertainties regarding infection status could act as a confounder in trajectory modeling. This highlights the value of our vaccine cohort for data analysis, as no participants experienced a COVID-19 infection during the one-year study period. This absence of infection-related confounding allows for a more accurate assessment of vaccine-induced humoral immune responses.

In the vaccine cohort, participants received inactivated SARS-CoV-2 vaccines, specifically BBIBP-CorV (Sinopharm) or CoronaVac (Sinovac Biotech), depending on vaccine availability. These vaccines contain pathogen antigens that stimulate the human immune system to produce corresponding antibodies. Both vaccines have been approved for emergency use by the World Health Organization (WHO) and were widely administered in China. As suggested by previous literature, the vaccine effectiveness (VE) estimates for BBIBP-CorV, CoronaVac, and combined vaccinations were similar (26).

Our study demonstrated that individuals with SARS-CoV-2 infection prior to the vaccine cohort experienced a significant increase in antibody levels after receiving the first dose of an inactivated SARS-CoV-2 vaccine. These levels remained relatively high for up to a year. In individuals without prior infection, a certain level of antibodies was sustained until one month after the second dose. A more robust humoral immune response was observed one month after the booster vaccination (dose 3) in individuals without prior infection. Furthermore, antibody titers remained at a relatively high level for up to six months after the booster dose. It was consistent with findings from previous studies on BBIBP-CorV or CoronaVac vaccination (10, 17, 27). The results highlighted the significance of a booster dose, particularly for individuals without prior infection.

By applying longitudinal K-means clustering to vaccine-induced humoral immune data for all participants in the vaccine cohort, we identified two distinct trajectories (two KML classes) for S-Igs and N-Igs titers. Further analysis revealed that preexisting infection status was the primary factor influencing these trajectories. This finding aligns with established evidence demonstrating divergent vaccine-elicited antibody titers between individuals with and without infection prior to vaccination, across both inactivated and mRNA vaccines (7–11, 28).

Regarding the heterogeneity of the humoral immune response, previous studies suggested differences in antibody responses across gender and age groups. Antibody responses to vaccines (both inactivated and mRNA vaccines) deteriorate with age and are frequently higher in females than males (11–13, 18, 19, 29–33). The relationship between the presence of chronic diseases and vaccine-induced antibody titers remains unclear. Some studies have identified chronic diseases as independent predictors of a low antibody response after vaccination (13, 17, 18), while others have shown no significant association (34). In our study, growth mixture model analysis indicated that male sex and advanced age were fixed effects associated with reduced antibody titers within each KML class, consistent with other research. However, our analysis revealed diabetes as a significant fixed-effect modifier of S-Igs titer trajectories, with individuals with diabetes showing higher antibody levels over time. It may be partially explained by the disproportionately higher prevalence of diabetes among individuals with prior infection compared to those without prior infection in the vaccine cohort (Table S1). Wong et al. (6) demonstrated that convalescent COVID-19 patients with high-persistent antibody titers were more likely to have chronic conditions (including diabetes).

Few studies have explored the subtypes of vaccine-induced humoral immune dynamics and their clinical implications. The only study we found with a comparable topic was by Wei et al. (14), who used latent class mixed models to analyze the dynamics of anti-spike antibody responses to natural SARS-CoV-2 infection in a population of 7,256 individuals from the United Kingdom (14). They identified a group of participants as “nonresponders”, characterized by the lack of anti-spike antibody development. Further logistic regression analysis revealed that individuals who were older and reported fewer symptoms during infection were more likely to belong to this group.

Our trajectory modeling identified five KML classes with distinct humoral response patterns among infection-naïve individuals. Among them, class A, characterized by minimal antibody titers and predominance in older adults, was similar to the “nonresponders” class in Wei et al. (14). However, being in class A was associated with an increased risk of prolonged recovery time, sore throat, and limb pain in subsequent infections. In contrast, class D (“high S-IgG booster responder”), marked by robust S-IgG amplification after the booster dose, tends to exhibit fewer symptoms and a lower likelihood of experiencing fever and fatigue. The clinical implications of these KML classes could aid in identifying individuals at varying levels of potential risk before the occurrence of the infection, enabling more targeted and precise immunization strategies. It may help transform passive antibody surveillance into an active precision public health tool, addressing critical gaps in pandemic preparedness.

Faro-Viana et al. (12) observed significant inter-individual variation in the amplitude and nature of the humoral response after a single dose of an mRNA vaccine, with age, sex, prior exposure, and medication use explaining only part of this variation. Our findings support this observation, suggesting that there may be underlying biological factors driving differences in immune trajectories. In a study by Bert et al. (2021), spike-binding antibody levels induced by SARS-CoV-2 vaccination were correlated with the expansion of spike- and RBD-specific memory B-cells across various vaccine platforms (35). Additionally, previous studies have shown that host genetic factors (such as specific HLA class II alleles) (36–39) and global features of the B-cell receptor (BCR) repertoire [66, 69] play crucial roles in vaccine-induced immunity. For example, Avnir et al. (2016) demonstrated that a phenylalanine (F)-to-leucine (L) polymorphism in the immunoglobulin heavy-chain variable locus (IGHV1-69) can modulate B-cell clonal expansion following H5N1 influenza vaccination [69]. Within each KML class, there may be shared public clonotypes, shared public antibodies, similar BCR clonal expansions (40), and other common genetic determinants.

Furthermore, while some research has monitored the durability of specific antibody levels after SARS-CoV-2 infection (3, 23, 41, 42), no concrete conclusions have been drawn about whether antibody titers correlate with protection from reinfection. Our study indicated that individuals with higher total antibody levels 15 days after discharge had a higher risk of reinfection, while those who had longer hospitalization/viral shedding duration had a lower reinfection risk. It may not be entirely robust due to the limitation of the sample size; however, there could be underlying mechanisms at play. Existing evidence has revealed a significantly longer duration of viral shedding in the asymptomatic group compared to the symptomatic group (43–45), alongside significantly lower serum levels of SARS-CoV-2-specific IgM, IgG, and IgA antibodies (46). These findings suggest that individuals identified at higher risk of reinfection might be those with mild symptoms and asymptomatic patients.

One limitation of the study is the small sample size of individuals with infection prior to the vaccine cohort, due to the low number of infected individuals in Sichuan Province prior to the initiation of the vaccination cohort. The association between reinfection risk and clinical data from previous infections needs to be further evaluated with more comprehensive data. Also, due to the limited sample size, we did not conduct subgroup analysis based on vaccine types. However, the vaccine effectiveness (VE) estimates for BBIBP-CorV and CoronaVac, as well as combined vaccinations, were similar (47), and our sensitivity analysis suggested that after adjusting for age, vaccine type did not significantly affect the trajectory clusters. Additionally, we solely measured binding antibodies against the spike (S) and nucleocapsid (N) proteins (S-Igs and N-Igs), while neutralizing antibodies and T-cell responses post-vaccination are also critical indicators of an individual’s vaccine-induced immunity. Furthermore, our study design has limitations in the variables collected, including the potential omission of socioeconomic factors, as well as the absence of genetic data and information on global features of the B-cell receptor (BCR) repertoire. To gain a deeper understanding of the underlying mechanisms, future study designs should focus on collecting and analyzing these missing variables to further explore the potential factors influencing immune response dynamics.

In conclusion, this study integrated multisource longitudinal data with trajectory modeling to delineate the heterogeneity of vaccine-induced humoral immune dynamics and their clinical implications for SARS-CoV-2 infection outcomes. Three principal findings emerge: First, preexisting infection status served as the dominant stratifier of antibody trajectory divergence across the cohort. Secondly, among individuals without prior infection before the vaccine cohort, we identified five distinct immune response patterns with clinical implications. The age-associated “minimal response” subtype (class A) was associated with an increased risk of prolonged recovery time, sore throat, and limb pain in subsequent infections. In contrast, the subtype characterized by a marked increase in S-Igs titers after the booster dose (class D) exhibited fewer symptoms, with a lower likelihood of experiencing fever and fatigue. It contributes to establishing the clinical implications of the identified KML classes. Third, for individuals with prior infection, reinfection susceptibility correlated inversely with post-discharge antibody titers and positively with initial infection viral shedding duration. Collectively, these findings enhance our understanding of the dynamics of vaccine-induced humoral response heterogeneity and how this heterogeneity can help predict clinical manifestations in subsequent infections. This will better assist in identifying vulnerable populations during epidemic surveillance and managing epidemics more effectively.

## MATERIALS AND METHODS

### Cohort design and participants

This study leveraged multisource data from Sichuan Province, China, with the vaccine cohort serving as its core component. This cohort, consisting of 39 individuals with prior SARS-CoV-2 infection and 166 individuals without prior SARS-CoV-2 infection, enrolled a total of 205 participants from Sichuan Province, China. It served as a key center within a multicenter prospective cohort study (10), adhering to uniform procedures and obtaining identical consent.

Enrollment was conducted between April 2021 and July 2021. Permanent residents aged ≥18 years who were willing to receive inactivated SARS-CoV-2 vaccine and to participate in the cohort follow-up were eligible. In addition to the general population, individuals with a history of natural infection, which was reviewed by the Centers for Disease Control and Prevention, were also included. We had access to clinical data, including follow-up information collected 15 days after discharge, from individuals with prior infections during their initial infection. The discharge criterion for SARS-CoV-2 patients was three consecutive negative nucleic acid tests, underscoring the reliability of the duration of hospitalization as an indicator of viral shedding duration.

Regarding the vaccination strategy in the vaccine cohort, all participants received their initial dose of inactivated SARS-CoV-2 vaccines upon enrollment, followed by the second dose administered approximately six weeks later. However, individuals without a prior infection received an additional booster dose approximately six months after the second vaccination. It is important to note that the vaccination administration was uniformly coordinated.

In general, all participants underwent a total of five successive follow-ups: baseline (before dose 1), ~6 weeks after dose 1 (before dose 2), one month after dose 2, six months after dose 2 (before dose 3 for the naïve), and 12 months after dose 2. For those without prior infection who received a booster vaccination, an additional follow-up was conducted one month after dose 3. Participants received either BBIBP-CorV (Sinopharm) or CoronaVac (Sinovac Biotech) based on vaccine availability. Of the 205 participants, 129 received BBIBP-CorV (Sinopharm) and 76 received CoronaVac (Sinovac Biotech). As suggested by previous literature, the vaccine effectiveness (VE) estimates for BBIBP-CorV and CoronaVac, as well as combined vaccinations, were similar (47).

In each follow-up, all participants completed a questionnaire regarding their extrinsic characteristics, including gender, age, smoking status, drinking status, exposure to secondhand smoke, and chronic disease status. Additionally, a venous blood sample was drawn from each participant to test the level of anti-spike protein pan-immunoglobulins (S-Igs) and anti-nucleocapsid protein pan-immunoglobulins (N-Igs).

Due to the close-contact management mode in China before December 2022 (48–50), outbreaks in the community in Chengdu were rare. Whenever outbreaks did occur, all close contacts underwent nucleic acid testing every 3 to 5 days to ensure they were not infected with COVID-19. Among these close contacts, only a small number were actually found to be infected with COVID-19. Specifically, among 40,425 close contacts from January 22, 2020, to March 1, 2022, in Chengdu, only 400 (0.99%) close contacts were converted into cases (48). The Chengdu Center for Disease Control and Prevention (CDC) confirmed that none of the participants was infected during the vaccine cohort follow-ups (from April 2021 to June 2022), based on close contact tracking and their nucleic acid test results.

**Fig 4:**
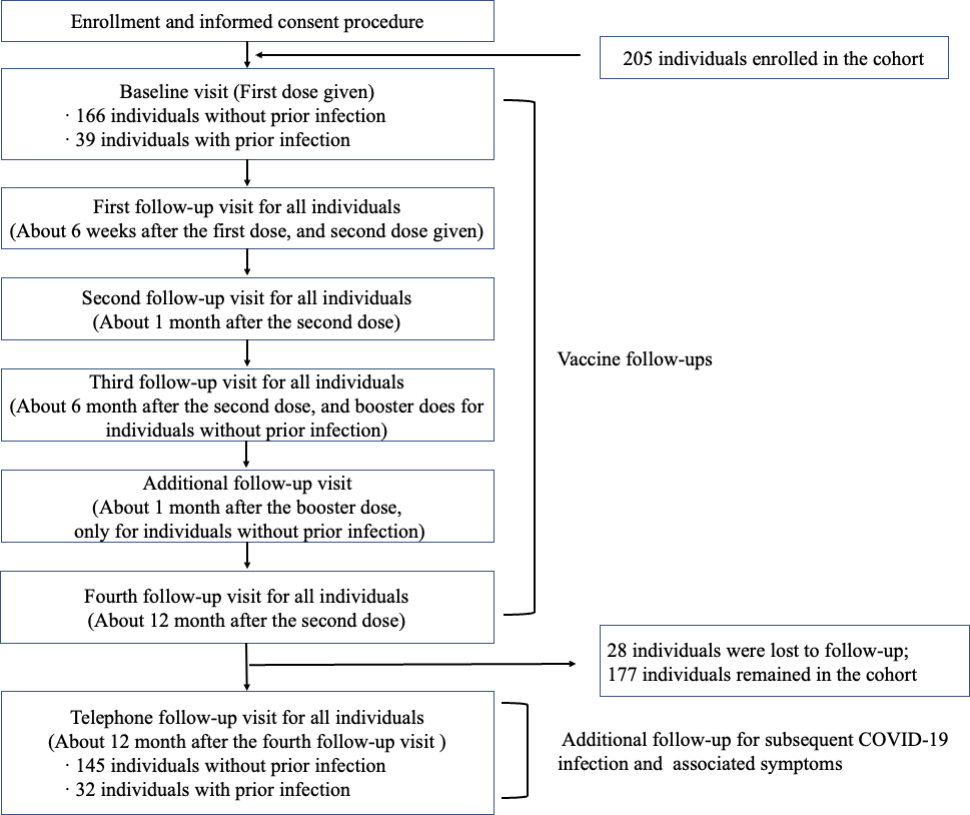
Study flowchart.

To assess their SARS-CoV-2 infection status over the subsequent year, an additional telephone follow-up was conducted in July 2023. This follow-up aimed to collect self-reported information on respondents’ infection status and associated symptoms since the previous follow-up. Determination of SARS-CoV-2 infection relied on inquiries about self-test antigen results and corresponding symptoms. Clinical data from previously infected patients were collected both during their hospitalization and in a follow-up conducted half a month after discharge. The whole follow-up schedule is shown in Fig. 4.

### Laboratory tests

As part of a multicenter cohort study (10), plasma was separated from the respondents’ venous blood samples within 4 hours of collection at the local public health clinical center, and all laboratory tests were conducted at the Christophe Mérieux Laboratory, National Institute of Pathogen Biology, Chinese Academy of Medical Sciences and Peking Union Medical College in Beijing, China. The respondents’ humoral immune response due to SARS‐CoV‐2 infection and COVID‐19 vaccination was evaluated by detecting the total binding antibodies against spike (S) and nucleocapsid (N) proteins (51). Plasma samples were inactivated at 56°C for 30 minutes and then tested for anti-spike protein pan-immunoglobulins (S-Igs) and anti-nucleocapsid protein pan-immunoglobulins (N-Igs) using electrochemiluminescence immunoassay kits following the manufacturer’s instructions (Roche Diagnostics).

### Statistical analysis

To visually depict the distribution of S‐Igs and N-Igs titers in the population across vaccine follow-ups, and to compare differences between individuals with and without natural infection, a descriptive analysis was conducted using violin plots and box plots. Two-tailed *t*-tests were applied to examine whether variations exist in the average levels of antibodies detected during each follow-up within the two groups.

Furthermore, to capture the dynamic pattern of antibody titers among individuals, trajectory methods were applied to the humoral response data collected during vaccine follow-ups. Partitional clustering (nonparametric algorithm) was implemented as the primary trajectory pattern detection framework. It was selected over parametric clustering approaches due to the absence of prior studies providing precise model specifications in this context. For partitional clustering, we employed K-means for joint longitudinal data to cluster the combined trajectories of S-Igs and N-Igs titers (52) and to determine the optimal cluster number with the Calinski–Harabasz index (53). To evaluate if demographic characteristics, health behaviors, chronic disease status, and infection history were related to the classification, *t*-tests and χ tests were displayed. The longitudinal cluster analysis was conducted using the kml3d package (version 2.4.6) within the R statistical environment (version 4.2.1).

For all participants, we cluster trajectories with data from the initial four follow-ups considering comparability, as both individuals with and without prior infection underwent identical administration protocols during these follow-ups. We initially applied K-means clustering to jointly analyze longitudinal trajectories of S-Igs and N-Igs titers, identifying two distinct temporal patterns. The clustering results revealed a robust association between trajectory patterns and prior infection status, providing empirical justification for specifying prior infection as the predictor of trajectory membership in subsequent parametric modeling. Leveraging this empirical foundation, a growth mixture model (GMM) was employed to further investigate the latent effects of additional factors, including age, gender, smoking and drinking status, exposure to secondhand smoke, and chronic diseases, on trajectory patterns. GMM is a finite mixture model (54). It estimates an average growth curve for each class, allowing for variations between individuals within the same class. Due to limited sample size, the trajectories of S-Igs and N-Igs titers were analyzed separately in the GMM model.

Given the distinct patterns of antibody titer trajectories between individuals with and without prior infection, as well as differences in their vaccination administration, a subgroup analysis was conducted. K-means for joint longitudinal data were applied to model the trajectories separately for individuals with and without prior infections. To assess the robustness of the identified KML classes, we validated them using PERMANOVA. PERMANOVA was employed to test whether the differences in antibody titer trajectories across the identified KML classes were statistically significant. It can be considered a multivariate extension of ANOVA. However, it makes no assumptions regarding the distribution of multivariate normality or homogeneity of variances. In PERMANOVA, variation within a group is directly calculated from a distance matrix, with Euclidean distance applied in this analysis. The pseudo-F statistic for the PERMANOVA test is calculated in the same manner as the conventional ANOVA F-statistic (55, 56).

Many of the naïve individuals in the vaccine cohort experienced their initial SARS-CoV-2 infection in the year following the vaccine follow-ups. To examine the associations between their KML class membership and the occurrence of symptoms during their initial infection, for those who reported being infected during the telephone follow-up, we applied logistic regression with stepwise variable selection based on the Akaike Information Criterion (AIC). This analysis adjusted for demographic variables and antibody titers at each follow-up. In these regression models, each trajectory class was treated as a dummy variable, with class B set as the reference category. Covariates included age, gender, recent alcohol consumption, recent smoking, recent exposure to secondhand smoke, and the presence of chronic diseases. The outcomes of interest were fatigue, anosmia, nasal obstruction, prolonged recovery time, fever, sore throat, and limb pain. Additionally, the correlation between the class of antibody titer trajectories and the number of symptoms experienced was examined using Poisson regression. In these regression models, demographic variables included age, gender, recent alcohol consumption, recent smoking, recent exposure to secondhand smoke, and the presence of chronic diseases.

To further identify predictors of elevated risk of reinfection post-SARS-CoV-2 among individuals with a history of natural infection, clinical data during their initial infection were considered using logistic regression with stepwise variable selection based on AIC.

For sensitivity analysis, we assessed whether different types of inactivated vaccines influenced the identification of trajectory clusters. Considering the effect of age on immune response (13), a multinomial logistic regression model, adjusted for age, was conducted to evaluate whether the type of inactivated vaccine had a significant effect on the trajectory clusters. In the multinomial logistic regression model, class B, which corresponds to the intermediate trajectory, was set as the reference class. Also, we investigated the associations with demographics and later infections for those with prior infection using Fisher’s exact test. We also examined how the symptoms associated during later infection correlated with one another using Phi coefficients.

All statistical analyses were performed using R version 4.2.1.

## Data Availability

Due to the inclusion of sensitive and confidential information, such as patient data, the data used in this research will not be shared publicly. However, anonymized individual-level data from the study can be made available to researchers who submit a methodologically sound proposal. Proposals should be directed to Yunhan Yu (yuyh22@tsinghua.org.cn).

## AFTER EPUB

*[This article was published on 11 August 2025 with errors in Tables 1 and 2. The tables were corrected in the current version, posted on 17 September 2025.]*

## SUPPLEMENTAL MATERIAL

Supplemental figures and tables spectrum.02191-24-s0001.docx Fig. S1 to S5 and Tables S1 to S21.

ASM does not own the copyrights to Supplemental Material that may be linked to, or accessed through, an article. The authors have granted ASM a non-exclusive, world-wide license to publish the Supplemental Material files. Please contact the corresponding author directly for reuse.
