# Supplementary material for: Humoral response dynamics following inactivated SARS-CoV-2 vaccination and their association with subsequent infection and symptoms in individuals with and without prior SARS-CoV-2 infection: evidence from Sichuan Province, China
Source: Microbiol Spectr. 2025 Aug 11;13(10):e02191-24. doi: 10.1128/spectrum.02191-24 (PMC12502705; doi:10.1128/spectrum.02191-24)
Supplement: Supplemental figures and tables — Fig. S1 to S5 and Tables S1 to S21. [file spectrum.02191-24-s0001.docx]

Supp. Table 1: Summary table of all the participants in the cohort

|  | Total  (N=205) | With past infection  (N=39) | Without past infection  (N=166) | p-Value |
| --- | --- | --- | --- | --- |
| Age  Median  IQR | 46  33,57 | 49  40,59.5 | 45  32,56 | 0.18 |
| Sex  Female  Male | 104 (50.7%)  101 (49.3%) | 19 (48.7%)  20 (51.3%) | 85 (51.2%)  81 (48.8%) | 0.86 |
| Highest education levels attained  Junior high or less  High school  Undergraduate or more | 77 (37.6%)  33 (16.1%)  95 (46.3%) | 21 (53.8%)  5 (12.8%)  13 (33.4%) | 56 (33.7%)  28 (16.9%)  82 (49.4%) | 0.04 |
| Recently smoke  No  Yes | 165 (80.5%)  40 (19.5%) | 33 (84.6%)  6 (15.4%) | 132 (79.5%)  34 (20.5%) | 0.73 |
| Recently drink  No  Yes | 159 (77.6%)  46 (22.4%) | 27 (71.8%)  11 (28.2%) | 131 (78.9%)  35 (21.1%) | 0.39 |
| Exposure to secondhand smoke  No  Yes | 125 (61.0%)  80 (39.0%) | 26 (66.7%)  13 (33.3%) | 99 (48.3%)  67 (32.7%) | 0.47 |
| Presence of NCD  No  Yes | 164 (80.0%)  41 (20.0%) | 18 (46.2%)  21 (53.8%) | 146 (88.0%)  20 (12.0%) | <0.001 |
| Hypertension  No  Yes | 186 (90.7%)  19 (9.3%) | 30 (76.9%)  9 (23.1%) | 156 (94.0%)  10 (6.0%) | 0.003 |
| Diabetes  No  Yes | 193 (94.1%)  12 (5.9%) | 32 (82.1%)  7 (17.9%) | 161 (97.0%)  5 (3.0%) | 0.002 |

Supp. Table 2: Tests result of demographic variables and two KML classes among all participants

| variable name | Test | p-value |
| --- | --- | --- |
| age | Kolmogorov-Smirnov test | 0.11 |
| gender | Chi-square test | 0.91 |
| previously infected | Chi-square test | <0.001 |
| Recently smoke | Chi-square test | 0.50 |
| Recently drink | Chi-square test | 0.72 |
| Exposure to secondhand smoke | Chi-square test | 0.62 |
| NCD | Chi-square test | <0.001 |
| Hypertension | Fisher's Exact Test | 0.01 |
| Diabetes | Fisher's Exact Test | 0.003 |

Supp. Table 3: Class-membership probabilities of the naïve individuals' trajectory pattern

| ID | Prob | ID | Prob | ID | Prob | ID | Prob |
| --- | --- | --- | --- | --- | --- | --- | --- |
| 1 | 1.00 | 41 | 1.00 | 81 | 0.93 | 121 | 1.00 |
| 2 | 0.98 | 42 | 0.83 | 82 | 1.00 | 122 | 1.00 |
| 3 | 1.00 | 43 | 0.98 | 83 | 1.00 | 123 | 1.00 |
| 4 | 0.61 | 44 | 1.00 | 84 | 1.00 | 124 | 0.97 |
| 5 | 1.00 | 45 | 0.98 | 85 | 1.00 | 125 | 0.75 |
| 6 | 1.00 | 46 | 0.81 | 86 | 1.00 | 126 | 1.00 |
| 7 | 0.92 | 47 | 0.49 | 87 | 0.61 | 127 | 1.00 |
| 8 | 1.00 | 48 | 0.58 | 88 | 0.54 | 128 | 1.00 |
| 9 | 1.00 | 49 | 1.00 | 89 | 0.89 | 129 | 1.00 |
| 10 | 1.00 | 50 | 1.00 | 90 | 1.00 | 130 | 1.00 |
| 11 | 1.00 | 51 | 1.00 | 91 | 1.00 | 131 | 0.62 |
| 12 | 1.00 | 52 | 0.99 | 92 | 0.72 | 132 | 1.00 |
| 13 | 0.61 | 53 | 0.71 | 93 | 1.00 | 133 | 1.00 |
| 14 | 1.00 | 54 | 0.99 | 94 | 1.00 | 134 | 1.00 |
| 15 | 1.00 | 55 | 1.00 | 95 | 0.99 | 135 | 1.00 |
| 16 | 1.00 | 56 | 1.00 | 96 | 1.00 | 136 | 1.00 |
| 17 | 1.00 | 57 | 1.00 | 97 | 0.98 | 137 | 0.88 |
| 18 | 1.00 | 58 | 1.00 | 98 | 1.00 | 138 | 1.00 |
| 19 | 1.00 | 59 | 1.00 | 99 | 1.00 | 139 | 1.00 |
| 20 | 1.00 | 60 | 1.00 | 100 | 1.00 | 140 | 0.74 |
| 21 | 1.00 | 61 | 0.96 | 101 | 1.00 | 141 | 1.00 |
| 22 | 0.85 | 62 | 1.00 | 102 | 1.00 | 142 | 0.51 |
| 23 | 0.91 | 63 | 1.00 | 103 | 1.00 | 143 | 0.73 |
| 24 | 1.00 | 64 | 0.99 | 104 | 0.99 | 144 | 1.00 |
| 25 | 1.00 | 65 | 1.00 | 105 | 0.90 | 145 | 0.97 |
| 26 | 1.00 | 66 | 1.00 | 106 | 1.00 | 146 | 1.00 |
| 27 | 1.00 | 67 | 0.94 | 107 | 0.97 | 147 | 0.95 |
| 28 | 0.59 | 68 | 0.90 | 108 | 0.99 | 148 | 1.00 |
| 29 | 0.86 | 69 | 1.00 | 109 | 1.00 | 149 | 1.00 |
| 30 | 0.99 | 70 | 0.99 | 110 | 1.00 | 150 | 1.00 |
| 31 | 1.00 | 71 | 0.68 | 111 | 1.00 | 151 | 1.00 |
| 32 | 1.00 | 72 | 0.81 | 112 | 0.60 | 152 | 1.00 |
| 33 | 1.00 | 73 | 1.00 | 113 | 1.00 | 153 | 0.99 |
| 34 | 0.99 | 74 | 1.00 | 114 | 0.98 | 154 | 0.99 |
| 35 | 1.00 | 75 | 1.00 | 115 | 0.72 | 155 | 0.87 |
| 36 | 0.88 | 76 | 0.94 | 116 | 1.00 | 156 | 0.98 |
| 37 | 1.00 | 77 | 1.00 | 117 | 1.00 | 157 | 1.00 |
| 38 | 0.98 | 78 | 1.00 | 118 | 1.00 | 158 | 1.00 |
| 39 | 0.83 | 79 | 1.00 | 119 | 0.92 | 159 | 0.50 |
| 40 | 0.97 | 80 | 1.00 | 120 | 1.00 | 160 | 1.00 |

Supp. Table 4: Demographic Characteristics of Individuals without a History of Infection, Stratified by KML Class

|  | Total  (N=166) ^1^ | Class A  (N=53) ^1^ | Class B  (N=39) ^1^ | Class C  (N=38) ^1^ | Class D  (N=21) ^1^ | Class E  (N=15) ^1^ | p-Value ^2^ |
| --- | --- | --- | --- | --- | --- | --- | --- |
| Age  Median  IQR | 45  32,56 | 53  43,62 | 41  31,54 | 41  31,54 | 39  32,47 | 40  35,53 | <0.01 |
| Sex  Female  Male | 84 (51%)  82 (49%) | 25 (47%)  28 (53%) | 21 (54%)  18 (46%) | 21 (55%)  17 (45%) | 9 (43%)  12 (57%) | 8 (53%)  7 (47%) | 0.86 |
| Smoking  No  Yes | 132 (80%)  34 (20%) | 41 (77%)  12 (23%) | 33 (85%)  6 (15%) | 32 (84%)  6 (16%) | 13 (62%)  8 (38%) | 13 (87%)  2 (13%) | 0.27 |
| Drinking  No  Yes | 131 (79%)  35 (21%) | 41 (77%)  12 (23%) | 33 (85%)  6 (15%) | 28 (74%)  10 (26%) | 16 (76%)  5 (24%) | 13 (87%)  2 (13%) | 0.75 |
| Diabetes  No  Yes | 161 (97%)  5 (3%) | 52 (98%)  1 (2%) | 38 (97%)  1 (3%) | 36 (95%)  2 (5%) | 20 (95%)  1 (5%) | 15(100%)  0 (0%) | 0.78 |
| Hypertension  No  Yes | 156 (94%)  10 (6%) | 51 (96%)  2 (4%) | 36 (92%)  3 (8%) | 35 (92%)  3 (8%) | 21(100%)  0 (0%) | 13(87%)  2 (13%) | 0.42 |
| ESHS  No  Yes | 99 (60%)  67 (40%) | 37 (70%)  16 (30%) | 21 (54%)  18 (46%) | 25 (66%)  13 (34%) | 7 (33%)  14 (67%) | 9 (60%)  6 (40%) | 0.06 |

* ^1^ n (%); Median (IQR); ^2^ Anova for age; Fisher's Exact Test for other category variables; The table includes all individuals without a history of infection in the vaccine cohort (N = 166)

Supp. Table 5: Comparison of Age Among Individuals in 5 Different KML Classes Using TUKEY Test in the Naïve Individuals

|  | Difference | adjusted p-value |
| --- | --- | --- |
| B-A | -9.32 | 0.01 |
| C-A | -9.12 | 0.02 |
| D-A | -10.92 | 0.02 |
| E-A | -7.92 | 0.29 |
| C-B | 0.21 | 1.00 |
| D-B | -1.59 | 0.99 |
| E-B | 1.41 | 1.00 |
| D-C | -1.80 | 0.99 |
| E-C | 1.20 | 1.00 |
| E-D | 3.00 | 0.97 |

Supp. Table 6: COVD-19 infection status among 5 KML classes in the naïve individuals during the outbreak in late December 2022

| Variable | Category | Infected | Not-infected |
| --- | --- | --- | --- |
| KML class | A | 37(88.1%) | 6(11.9%) |
|  | B | 35(100%) | 0 |
|  | C | 26(83.9%) | 5(16.1%) |
|  | D | 17(89.5%) | 2(10.5%) |
|  | E | 12(100%) | 0 |

Supp. Table 7: Predictors' effects on having more symptoms during the first COVD-19 infection among the naïve individuals

| Variable | Category | OR | 95%CI | p-value |
| --- | --- | --- | --- | --- |
| KML class | A | - | - | - |
|  | B | - | - | - |
|  | C | -0.29 | (-0.54,-0.04) | 0.02 |
|  | D | -0.44 | (-0.79,-0.08) | 0.02 |
|  | E | - | - | - |
| S-Igs FU2 |  | 0.02 | (0,0.03) | 0.01 |
| N-Igs FU5 |  | 0 | (0,0) | 0.04 |

*: *SHS stands for Secondhand Smoke; FU is an abbreviation for follow-up, used to indicate the specific follow-up visit during which antibody titers were measured; and hyphens indicate that this specific KML class was not selected for the model, as stepwise variable selection was applied using the Akaike Information Criterion (AIC).*

Supp. Table 8: Predictors' effects on experiencing rather long recovery time

during the first COVD-19 infection among the naïve individuals

| Variable | Category | OR | 95%CI | p-value |
| --- | --- | --- | --- | --- |
| KML class | A | 9.298 | (2.258,38.294) | 0.002 |
|  | B | - | - | - |
|  | C | - | - | - |
|  | D | 0.126 | (0.010,1.644) | 0.114 |
|  | E | - | - | - |
| S-Igs FU4 |  | 1.002 | (1,1.005) | 0.051 |
| S-Igs FU5 |  | 1.001 | (1,1.002) | 0.013 |
| N-Igs FU5 |  | 1.011 | (1.002,1.019) | 0.015 |
| Recently drink | No | Ref |  |  |
|  | Yes | 0.387 | (0.112,1.343) | 0.135 |

*: *SHS stands for Secondhand Smoke; FU is an abbreviation for follow-up, used to indicate the specific follow-up visit during which antibody titers were measured; and hyphens indicate that this specific KML class was not selected for the model, as stepwise variable selection was applied using the Akaike Information Criterion (AIC).*

Supp. Table 9: Predictors' effects on having fever

during the first COVD-19 infection among the naïve individuals

| Variable | Category | OR | 95%CI | p-value |
| --- | --- | --- | --- | --- |
| KML class | A | - | - | - |
|  | B | - | - | - |
|  | C | - | - | - |
|  | D | 0.01 | (0,0.39) | 0.02 |
|  | E | 0.13 | (0.01,1.15) | 0.07 |
| S-Igs FU2 |  | 1.17 | (0.97,1.41) | 0.11 |
| S-Igs FU3 |  | 0.99 | (0.99,1) | 0.01 |
| N-Igs FU6 |  | 1.05 | (1.02,1.08) | 0 |
| age |  | 0.96 | (0.92,0.99) | 0.03 |
| Recently drink | No | Ref |  |  |
|  | Yes | 0.32 | (0.09,1.13) | 0.08 |

*: *SHS stands for Secondhand Smoke; FU is an abbreviation for follow-up, used to indicate the specific follow-up visit during which antibody titers were measured; and hyphens indicate that this specific KML class was not selected for the model, as stepwise variable selection was applied using the Akaike Information Criterion (AIC).*

Supp. Table 10: Predictors' effects on having a sore throat

during the first COVD-19 infection among the naïve individuals

| Variable | Category | OR | 95%CI | p-value |  |
| --- | --- | --- | --- | --- | --- |
| KML class | A | 7.53 | (1.75,32.37) | 0.01 |  |
|  | B | - | - | - |  |
|  | C | - | - | - |  |
|  | D | - | - | - |  |
|  | E | - | - | - |  |
| N-Igs FU5 |  | 1.03 | (1.01,1.04) | 0.01 |  |
| N-Igs FU6 |  | 0.97 | (0.95,0.99) | 0.01 |  |

*: *SHS stands for Secondhand Smoke; FU is an abbreviation for follow-up, used to indicate the specific follow-up visit during which antibody titers were measured; and hyphens indicate that this specific KML class was not selected for the model, as stepwise variable selection was applied using the Akaike Information Criterion (AIC).*

Supp. Table 11: Predictors' effects on experiencing fatigue

during the first COVID-19 infection among naive individuals

| Variable | Category | OR | 95%CI | p-value |
| --- | --- | --- | --- | --- |
| KML class | A | - | - | - |
|  | B | - | - | - |
|  | C | - | - | - |
|  | D | 0.17 | (0.04,0.77) | 0.02 |
|  | E | - | - | - |
| N-Igs FU5 |  | 1.01 | (1,1.02) | 0.06 |

*: *SHS stands for Secondhand Smoke; FU is an abbreviation for follow-up, used to indicate the specific follow-up visit during which antibody titers were measured; and hyphens indicate that this specific KML class was not selected for the model, as stepwise variable selection was applied using the Akaike Information Criterion (AIC).*

Supp. Table 12: Predictors' effects on pain in the limbs

during the first COVID-19 infection among naive individuals

| Variable | Category | OR | 95%CI | p-value |
| --- | --- | --- | --- | --- |
| KML class | A | 6.61 | (1.13,38.83) | 0.04 |
|  | B | - | - | - |
|  | C | - | - | - |
|  | D | - | - | - |
|  | E | - | - | - |
| S-Igs FU3 |  | 1 | (1,1.01) | 0.03 |
| S-Igs FU4 |  | 1 | (0.99,1) | 0.16 |

*: *SHS stands for Secondhand Smoke; FU is an abbreviation for follow-up, used to indicate the specific follow-up visit during which antibody titers were measured; and hyphens indicate that this specific KML class was not selected for the model, as stepwise variable selection was applied using the Akaike Information Criterion (AIC).*

Supp. Table 13: Predictors' effects on anosmia

during the first COVID-19 infection among naive individuals

| Variable | Category | OR | 95%CI | p-value |
| --- | --- | --- | --- | --- |
| S-Igs FU2 |  | 1.24 | (1.02,1.51) | 0.04 |
| S-Igs FU3 |  | 1.00 | (0.99,1) | 0.2 |
| S-Igs FU4 |  | 1.00 | (1,1.01) | 0.02 |
| N-Igs FU3 |  | 1.04 | (1,1.09) | 0.07 |
| N-Igs FU4 |  | 0.73 | (0.48,1.13) | 0.17 |
| Exposure to SHS | No | Ref |  |  |
|  | Yes | 0.20 | (0.03,1.29) | 0.09 |
| Gender | Male | Ref |  |  |
|  | Female | 0.15 | (0.02,0.91) | 0.04 |

*: *SHS stands for Secondhand Smoke; FU is an abbreviation for follow-up, used to indicate the specific follow-up visit during which antibody titers were measured; and hyphens indicate that this specific KML class was not selected for the model, as stepwise variable selection was applied using the Akaike Information Criterion (AIC).*

Supp. Table 14: Predictors' effects on nasal obstruction

during the first COVID-19 infection among naive individuals

| Variable | Category | OR | 95%CI | p-value |
| --- | --- | --- | --- | --- |
| S-Igs FU3 |  | 0.99 | (0.98,1) | 0.03 |
| S-Igs FU6 |  | 1 | (1,1) | 0.04 |
| N-Igs FU5 |  | 1.02 | (1.01,1.04) | 0 |
| N-Igs FU6 |  | 0.98 | (0.95,1) | 0.02 |

*: *SHS stands for Secondhand Smoke; FU is an abbreviation for follow-up, used to indicate the specific follow-up visit during which antibody titers were measured; and hyphens indicate that this specific KML class was not selected for the model, as stepwise variable selection was applied using the Akaike Information Criterion (AIC).*

Supp. Table 15.1: Demographic Characteristics of Individuals with a History of Infection, Stratified by KML Class

|  | Total  (N=39) ^1^ | Class A  (N=17) ^1^ | Class B  (N=15) ^1^ | Class C  (N=7) ^1^ | p-Value^2^ |
| --- | --- | --- | --- | --- | --- |
| Age  Median  IQR | 49  40,60 | 44  37,57 | 56  48,69 | 49  47,50 | 0.30 |
| Sex  Female  Male | 19 (49%)  20 (51%) | 7 (41%)  10 (59%) | 8 (53%)  7 (47%) | 5 (71%)  2 (29%) | 0.49 |
| Recently Smoke  No  Yes | 33 (85%)  6 (15%) | 15 (88%)  2 (12%) | 13 (87%)  2 (13%) | 5 (71%)  2 (29%) | 0.62 |
| Recently Drink  No  Yes | 28 (72%) 11 (28%) | 13 (76%)  4 (24%) | 11 (73%)  4 (27%) | 4 (57%)  3 (43%) | 0.59 |
| Presence of Diabetes  No  Yes | 32 (82%)  7 (18%) | 14 (82%)  3 (18%) | 12 (80%)  3 (20%) | 6 (86%)  1 (14%) | 0.99 |
| Presence of Hypertension  No  Yes | 30 (77%)  9 (23%) | 15 (88%)  2 (12%) | 10 (67%)  5 (33%) | 5 (71%)  2 (29%) | 0.33 |
| Exposure to Secondhand Smoke  No  Yes | 26 (67%)  13 (33%) | 11 (65%)  6 (35%) | 11 (73%)  4 (27%) | 4 (57%)  3 (43%) | 0.74 |

* ^1^ n (%); Median (IQR); ^2^ Anova for age; Fisher's Exact Test for other category variables; The table includes all individuals with a history of infection in the vaccine cohort (N = 39)

Supp. Table 15.2: Demographic Characteristics of Individuals with a History of Infection, Stratified by Their Reinfection Status

|  | Total  (N=32) ^1^ | Without reinfection  (N=15) ^1^ | With reinfection  (N=17) ^1^ | p-Value^2^ |
| --- | --- | --- | --- | --- |
| Age  Median  IQR | 49  37,55 | 51  47,58 | 44  35,50 | 0.26 |
| Sex  Female  Male | 18 (56%)  14 (44%) | 6 (40%)  9 (60%) | 12 (71%)  5 (29%) | 0.08 |
| Recently Smoke  No  Yes | 28 (88%)  4 (13%) | 12 (80%)  3 (20%) | 16 (94%)  1 (6%) | 0.25 |
| Recently Drink  No  Yes | 25 (78%)  7 (22%) | 11 (73%)  4 (27%) | 14 (82%)  3 (18%) | 0.68 |
| Presence of Diabetes  No  Yes | 26 (81%)  6 (19%) | 11 (73%)  4 (27%) | 15 (88%)  2 (12%) | 0.38 |
| Presence of Hypertension  No  Yes | 25 (78%)  7 (22%) | 11 (73%)  4 (27%) | 14 (82%)  3 (18%) | 0.68 |
| Exposure to Secondhand Smoke  No  Yes | 22 (69%)  10 (31%) | 11 (73%)  4 (27%) | 11 (65%)  6 (35%) | 0.71 |
| KML Class  A  B  C | 14 (44%)  11 (34%)  7 (22%) | 5 (33%)  6 (40%)  4 (27%) | 9 (53%)  5 (29%)  3 (18%) | 0.54 |

* ^1^ n (%); Median (IQR); ^2^ t-test for age; Fisher's Exact Test for other category variables; The table includes individuals with a history of infection in the vaccine cohort who responded to the telephone follow-up (N = 32).

Supp. Table 16: Demographic characteristics, clinical testing indicators during the initial infection, reinfection status, and associated symptoms in reinfection of three KML classes among those who previously infected

| **Characteristic** | **A**, N = 11^1^ | **B**, N = 7^1^ | **C**, N = 5^1^ | **p-value**^2^ |
| --- | --- | --- | --- | --- |
| Duration of hospitalization | 19 (15, 24) | 32 (29, 35) | 27 (26, 29) | 0.06 |
| Admission CD3+ and CD4+ count | 430 (313, 561) | 622 (396, 735) | 612 (557, 642) | 0.22 |
| CD3+ and CD4+ count 15 days after discharge | 471 (411, 540) | 432 (368, 525) | 534 (534, 547) | 0.84 |
| Admission CD3+ and CD8+ count | 242 (202, 409) | 463 (292, 519) | 316 (270, 466) | 0.32 |
| CD3+ and CD8+ count 15 days after discharge | 374 (303, 500) | 476 (213, 627) | 268 (204, 393) | 0.47 |
| Change of CD3+ and CD8+ count during hospitalization | 182 (168, 227) | 339 (136, 454) | 192 (173, 238) | 0.68 |
| Admission CD19+ count | 123 (88, 130) | 125 (107, 130) | 128 (128, 130) | 0.63 |
| CD19+ count 15 days after discharge | 125 (98, 140) | 152 (98, 199) | 137 (132, 179) | 0.69 |
| Admission CD56+ count | 113 (84, 142) | 92 (78, 129) | 164 (96, 184) | 0.30 |
| CD56+ count 15 days after discharge | 127 (77, 166) | 185 (64, 201) | 123 (109, 142) | >0.90 |
| Admission total antibody level | 50 (19, 86) | 0 (0, 37) | 40 (28, 90) | 0.21 |
| Total antibody level 15 days after discharge | 146 (120, 415) | 118 (109, 254) | 71 (50, 122) | 0.09 |
| Change in total antibody level | 241 (192, 517) | 211 (190, 231) | 178 (141, 263) | 0.29 |
| Had dose 3 vaccination |  |  |  | 0.41 |
| FALSE | 7 (64%) | 2 (29%) | 3 (60%) |  |
| TRUE | 4 (36%) | 5 (71%) | 2 (40%) |  |
| Had NCD |  |  |  | 0.66 |
| FALSE | 6 (55%) | 2 (29%) | 2 (40%) |  |
| TRUE | 5 (45%) | 5 (71%) | 3 (60%) |  |
| reinfection |  |  |  | 0.54 |
| FALSE | 5 (45%) | 5 (71%) | 4 (80%) |  |
| TRUE | 6 (55%) | 2 (29%) | 1 (20%) |  |
| Recovery time for reinfection |  |  |  | 0.74 |
| 0 | 5 (45%) | 5 (71%) | 4 (80%) |  |
| 1 | 1 (9.1%) | 0 (0%) | 0 (0%) |  |
| 3 | 2 (18%) | 1 (14%) | 0 (0%) |  |
| 7 | 3 (27%) | 0 (0%) | 1 (20%) |  |
| 8 | 0 (0%) | 1 (14%) | 0 (0%) |  |
| Had fever during reinfection |  |  |  | 0.81 |
| FALSE | 7 (64%) | 6 (86%) | 4 (80%) |  |
| TRUE | 4 (36%) | 1 (14%) | 1 (20%) |  |
| Coughed during reinfection |  |  |  | >0.90 |
| FALSE | 3 (50%) | 1 (50%) | 1 (100%) |  |
| TRUE | 3 (50%) | 1 (50%) | 0 (0%) |  |
| Had nasal obstruction during reinfection |  |  |  | 0.40 |
| FALSE | 4 (67%) | 2 (100%) | 0 (0%) |  |
| TRUE | 2 (33%) | 0 (0%) | 1 (100%) |  |
| Had a sore throat during reinfection |  |  |  | 0.65 |
| FALSE | 2 (33%) | 1 (50%) | 1 (100%) |  |
| TRUE | 4 (67%) | 1 (50%) | 0 (0%) |  |
| Had a running nose during reinfection |  |  |  | 0.64 |
| FALSE | 3 (50%) | 2 (100%) | 1 (100%) |  |
| TRUE | 3 (50%) | 0 (0%) | 0 (0%) |  |
| Anosmia during reinfection |  |  |  | >0.90 |
| FALSE | 5 (83%) | 2 (100%) | 1 (100%) |  |
| TRUE | 1 (17%) | 0 (0%) | 0 (0%) |  |
|  |  |  |  |  |
| Being fatigue during reinfection |  |  |  | >0.90 |
| FALSE | 3 (50%) | 1 (50%) | 0 (0%) |  |
| TRUE | 3 (50%) | 1 (50%) | 1 (100%) |  |
| Pain in the limbs during reinfection |  |  |  | 0.27 |
| FALSE | 5 (83%) | 2 (100%) | 0 (0%) |  |
| TRUE | 1 (17%) | 0 (0%) | 1 (100%) |  |
| ^1^Median (IQR); n (%) | | | | |
| ^2^Kruskal-Wallis rank sum test; Fisher's exact test | | | | |

Supp. Table 17: Impact of Vaccination with CoronaVac (Sinovac Biotech) vs. BBIBP-CorV (Sinopharm) on the Identification of Five KML Classes among the Naïve Individuals

| Variable | Category | OR | 95%CI | p-value |
| --- | --- | --- | --- | --- |
| KML class | A | 0.49 | (0.16,1.11) | 0.08 |
|  | B | Reference | - | - |
|  | C | 0.48 | (0.38,2.48) | 0.94 |
|  | D | 0.57 | (0.93,8.81) | 0.07 |
|  | E | 0.66 | (0.48,6.31) | 0.40 |

Supp. Table 18: PERMANOVA Analysis of Antibody Titer Trajectories by Five KML Class among Individuals with Prior Infection

| Source of variation | Sum of Squares | R2 | p-value |
| --- | --- | --- | --- |
| Model | 215,073,429 | 0.627 | 0.001 |
| Residual | 127,953,900 | 0.373 |  |
| Total | 343,027,329 | 1.000 |  |

Supp. Table 19: PERMANOVA Analysis of Antibody Titer Trajectories by Three KML Class among Individuals without Prior Infection

| Source of variation | Sum of Squares | R2 | p-value |
| --- | --- | --- | --- |
| Model | 5,138,867 | 0.064 | 0.124 |
| Residual | 74,864,427 | 0.936 |  |
| Total | 80,003,294 | 1.000 |  |

Supp. Table 20**:** Correlation Matrix of Self-Reported Symptoms in Individuals Infected After Vaccine Cohort Follow-Up (N=149)

|  | Have fever | Sore throat | Fatigue | Pain in the limbs | Long recovery time |
| --- | --- | --- | --- | --- | --- |
| Have fever | 1.00 | -0.02 | 0.20 | 0.18 | 0.03 |
| Sore throat | -0.02 | 1.00 | 0.00 | 0.08 | 0.01 |
| Fatigue | 0.20 | 0.00 | 1.00 | 0.17 | 0.03 |
| Pain in the limbs | 0.18 | 0.08 | 0.17 | 1.00 | 0.08 |
| Long recovery time | 0.03 | 0.01 | 0.03 | 0.08 | 1.00 |

Supp. Table 21**:** Comparative Demographic Characteristics of the Full Cohort and Supplementary Telephone Follow-Up Completers

|  | Total  (N=205) ^1^ | Telephone follow-up  (N=177) ^1^ | p-Value^2^ |
| --- | --- | --- | --- |
| Age  Median  IQR | 46  33,57 | 45  32,55 | 0.47 |
| Sex  Female  Male | 104 (51%)  101 (49%) | 83 (47%)  94 (53%) | 0.58 |
| Recently smoke  No  Yes | 165 (80%)  40 (20%) | 143 (81%)  34 (19%) | 0.94 |
| Recently drink  No  Yes | 159 (77.6%)  46 (22.4%) | 138 (78%)  39 (21%) | 0.92 |
| Exposure to secondhand smoke  No  Yes | 125 (61%)  80 (39%) | 108 (61%)  69 (39%) | >0.99 |
| Hypertension  No  Yes | 186 (91%)  19 (9%) | 162 (92%)  15 (8%) | 0.79 |
| Diabetes  No  Yes | 193 (94%)  12 (6%) | 167 (94%)  10 (6%) | 0.93 |

* ^1^ n (%); Median (IQR); ^2^ t-test for age; Fisher's Exact Test for other category variables

Supp. Figure 1: Correlation matrix among infected individuals' clinical variable during their first infection (N=39)


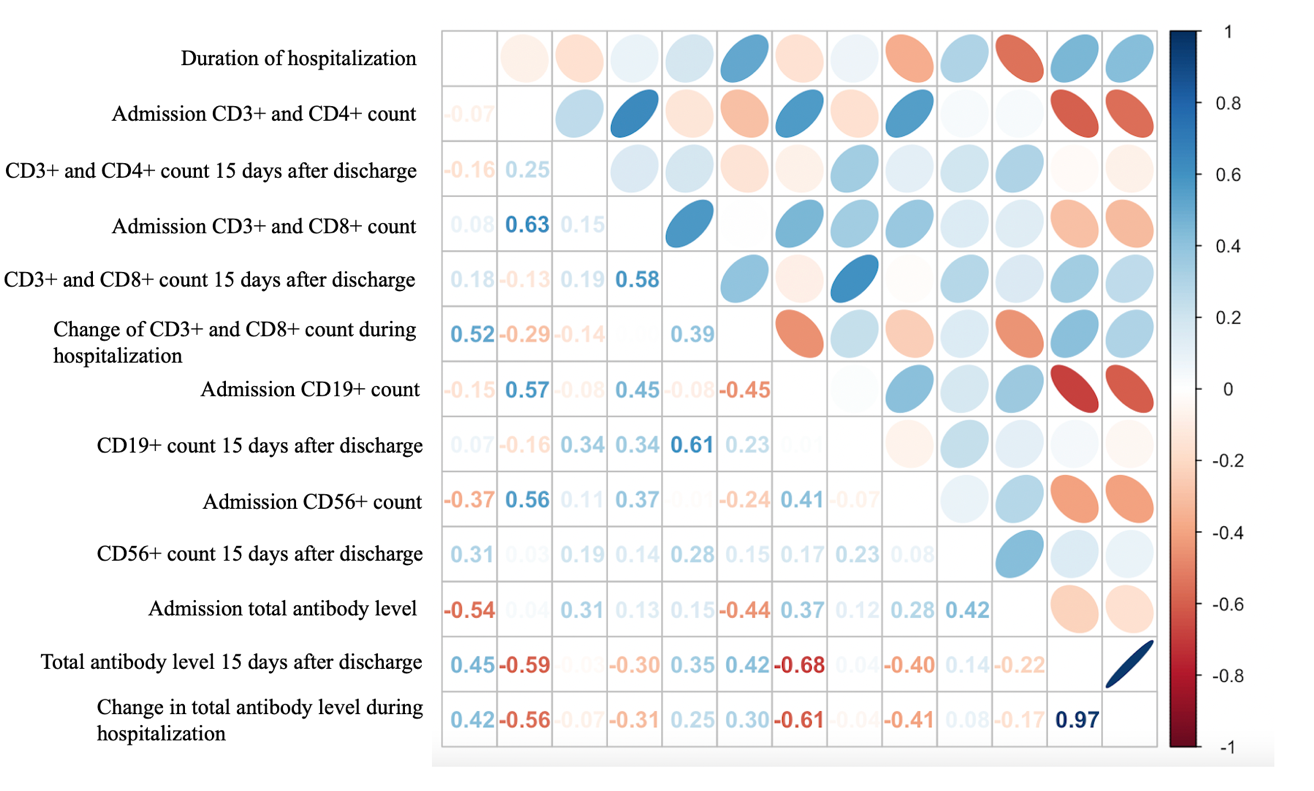


Supp. Figure 2: Calinski-Harabasz Index Dynamics Across Rerolling Iterations for Optimal Cluster Selection in Longitudinal K-Means Clustering among all 205 individuals


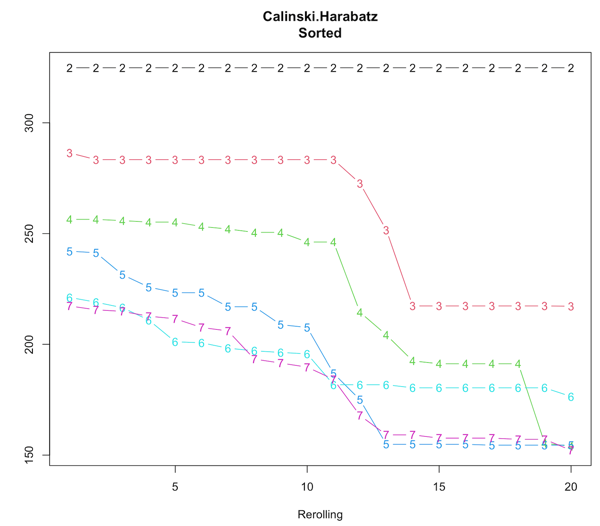


Note: Rerolling iterations represent the number of repeated cluster centroid reinitializations. Higher Calinski-Harabasz values indicate better cluster separation.

Supp. Figure 3: Mean Trajectories of Individuals With and Without Prior Infection Across the First Four Follow-Ups (N=205)


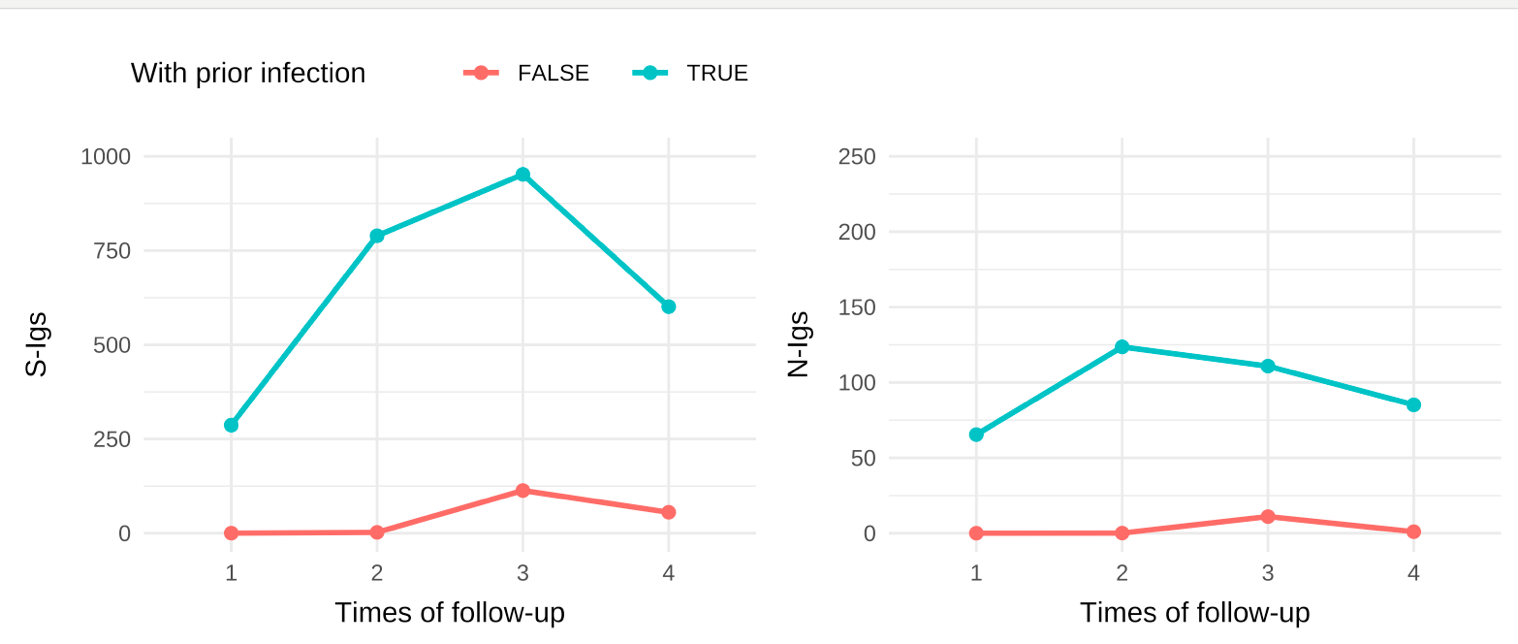


Supp. Figure 4: Calinski-Harabasz Index Dynamics Across Rerolling Iterations for Optimal Cluster Selection in Longitudinal K-Means Clustering among individuals without prior infection (N=166)


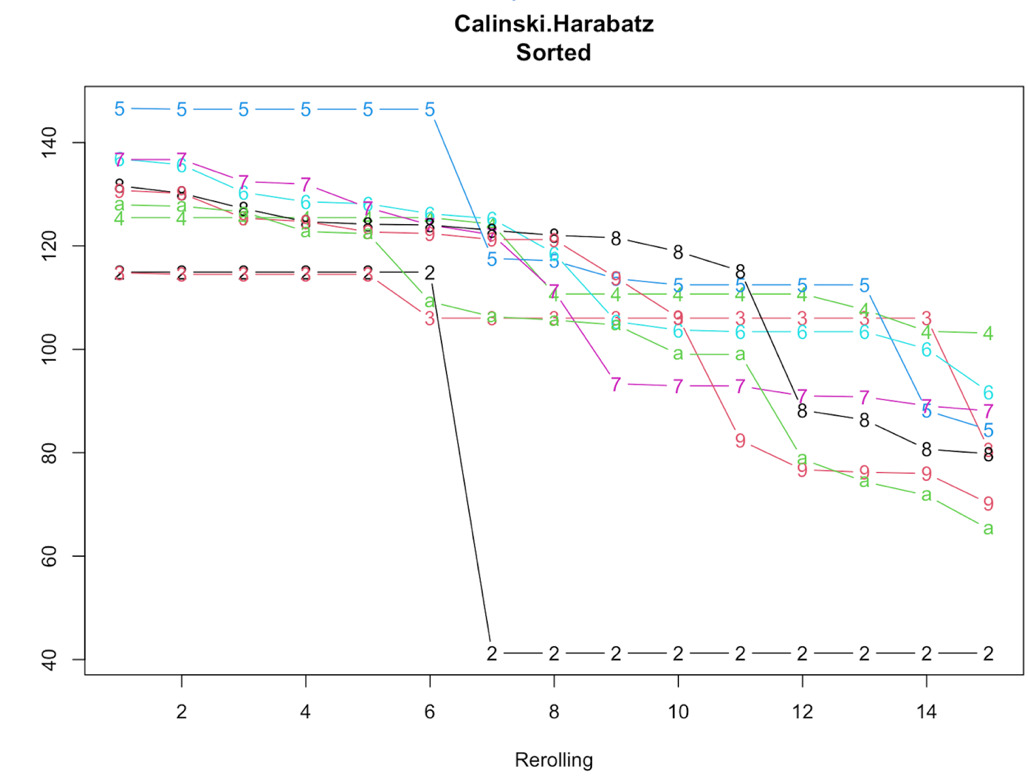


Note: Rerolling iterations represent the number of repeated cluster centroid reinitializations. Higher Calinski-Harabasz values indicate better cluster separation.

Supp. Figure 5: Calinski-Harabasz Index Dynamics Across Rerolling Iterations for Optimal Cluster Selection in Longitudinal K-Means Clustering among individuals with prior infection (N=39)


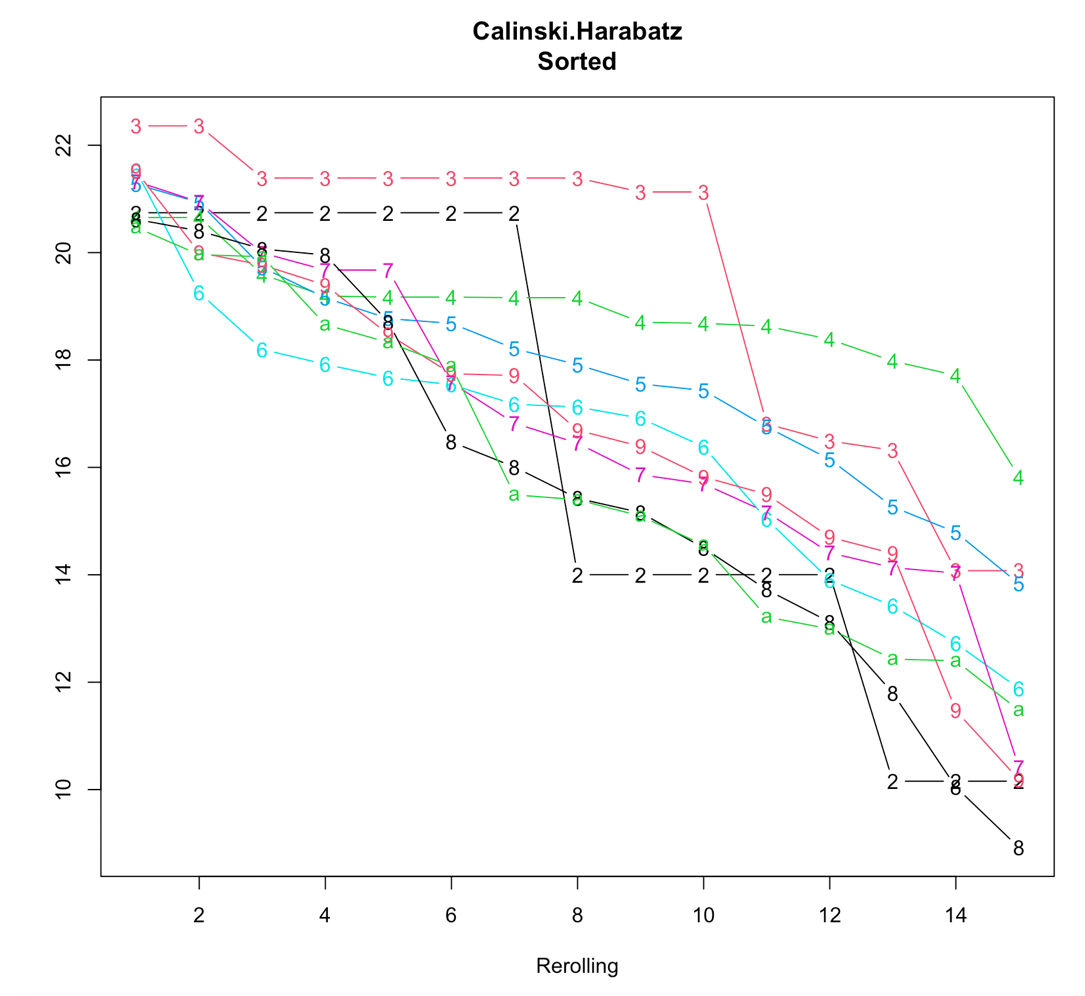


Note: Rerolling iterations represent the number of repeated cluster centroid reinitializations. Higher Calinski-Harabasz values indicate better cluster separation.
